# The Comparison of Sorption and Solubility Behavior of Four Different Resin Luting Cements in Different Storage Media

**Published:** 2016-06

**Authors:** Rashin Giti, Mahroo Vojdani, Jaafar Abduo, Rafat Bagheri

**Affiliations:** 1 Dept. of Prosthodontics, School of Dentistry, Shiraz University of Medical Sciences, Shiraz, Iran.; 2 Dept. of Prosthodontics and Biomaterials Research Centre, School of Dentistry, Shiraz University of Medical Sciences, Shiraz, Iran.; 3 Dept. of Restorative Dentistry, Melbourne Dental School, Melbourne University, Victoria, Australia.; 4 Dept. of Dental Materials and Biomaterials Research Centre, School of Dentistry, Shiraz University of Medical Sciences, Shiraz, Iran.

**Keywords:** Storage Media, Sorption and Solubility, Resin Luting Cements

## Abstract

**Statement of the Problem:**

Structural integrity and dimensional stability are the key factors that determine the clinical success and durability of luting cements in the oral cavity. Sorption and solubility of self-adhesive resin luting cements in food-simulating solutions has not been studied sufficiently.

**Purpose:**

This study aimed to compare the sorption and solubility of 2 conventional and 2 self-adhesive resin-based luting cements immersed in four different storage media.

**Materials and Method:**

A total of 32 disc-shaped specimens were prepared from each of four resin luting cements; seT (SDI), Panavia F (Kuraray), Clearfil SA Cement (Kuraray), and Choice 2 (Bisco). Eight specimens of each material were immersed in all tested solutions including n-heptane 97%, distilled water, apple juice, or Listerine mouth wash. Sorption and solubility were measured by weighing the specimens before and after immersion and desiccation. Data were analyzed by SPSS version 18, using two-way ANOVA and Tukey’s HSD test with *p*≤ 0.05 set as the level of significance.

**Results:**

There was a statistically significant interaction between the materials and solutions. The effect of media on the sorption and solubility was material-dependent. While seT showed the highest values of the sorption in almost all solutions, Choice 2 showed the least values of sorption and solubility. Immersion in apple juice caused more sorption than other solutions (*p*≤ 0.05).

**Conclusion:**

The sorption and solubility behavior of the studied cements were significantly affected by their composition and the storage media. The more hydrophobic materials with higher filler content like Choice 2 resin cement showed the least sorption and solubility. Due to their lower sorption and solubility, these types of resin-based luting cements are recommended to be used clinically.

## Introduction

Various adhesive cements are being frequently used for cementation of indirect restorations. The use of resin cements facilitates the application of more conservative restorations such as porcelain inlays, veneers and resin-retained fixed dental prostheses.[1] Resin luting cements have the advantage of superior mechanical and physical properties when compared to the traditional luting cements.[2] Resin cements are classified in 3 groups of conventional (total-etch), self-etch, and self-adhesive resin cements. One of the disadvantages of using conventional resin luting cements is their multi-step application which renders their quality for being technique-sensitive.[3]To overcome this problem, the new self-etch and self-adhesive luting cements are easier to use and require less clinical steps. This is owing to their composition which consists of monomers that have bonding as well as mineralizing capacities. 

The clinical success and durability of luting cements in the oral cavity depend on different properties such as structural integrity and dimensional stability which are functions of water sorption and solubility.[4]When resin cements are exposed to moist conditions, substances such as unreacted monomers dissolve from the cements, where the lost mass is measured as solubility.[4] Sorption arising from the polymer matrix hydrolytically degrades the network structure, debonds the silanized filler and consequently influences the solubility of these materials.[4] The solubility behavior of resins is related to several factors such as unreacted monomers, size and chemical compositions of material, and chemical characteristics of the solvent.[3] In the oral cavity, restorations are often close to the gingival crevice and in contact with the oral fluids. Therefore, water sorption and solubility of these materials may have unwanted consequences during clinical use including degradation of the cement which can lead to fracture of the restoration,[2] marginal leakage, and the risk of secondary caries.[5]Water sorption of a polymer mainly occurs in the resin matrix,[6] and the absorbed water acts as a plasticizer and leads to degradation of filler-matrix interface, material discoloration, and aesthetic issues in the restoration.[7] Moreover, solubility produces toxic substances such as formaldehyde and methacrylic acid. Accumulation of these products along with the residual monomers, fillers, and residual activators due to the polymerization can be hazardous to the oral soft tissues.[8]

Many studies have been conducted to evaluate the effect of different media on the physical and mechanical properties of resin-based materials.[4, 9-10] Yet, water sorption and solubility of self-adhesive resin luting cements in food-simulating solutions has not been widely studied. The aim of this study, therefore, was to compare the sorption and solubility of four adhesive resin cements in food-simulating solutions and Listerine mouth wash. The null hypothesis is that there is no difference between the conventional and self-adhesive resin cements in relation to sorption and solubility. 

## Materials and Method

Table 1 shows the description of all resin luting cements used in this study. For each type of cement, 32 discs-shaped specimens were prepared using a polyethylene mold of 10-mm diameter and 1-mm thickness. The cements were placed in the mold and pressed between two plastic matrix strips and glass slabs under hand pressure to extrude excess material and porosities. The top glass slab was removed, and the cement was cured according to the manufacturer’s instruction with an LED curing light at a wavelength range of 440-480nm and an emitting light intensity of 1500 mW/cm^2^ (Radi plus LED; SDI, Melbourne, Vic., Australia).

**Table 1:** Description of all the cements and solutions used in this study

**Name**	**Manufacturer**	**Type**	**Resin matrix**	**Filler content** **(Wt %) and type**	**Lot number**
seT	SDI, Victoria, Australia	Self-adhesive	MPE, UDMA Photo initiator	67% FAS glass Pyrogenic silica	S1209197
Panavia F	Kuraray Medical Inc., Okayama Japan	Conventional	HEMA, MDP, 5-NMSA	78% silanized silica, silanized colloidal silica, Silanized barium glass	051124
Clearfil SA Cement	Kuraray Noritake Dental Inc., Okayama, Japan	Self-adhesive	Bis-GMA, TEG-DMA, MDP	78% Silanized barium glass, silanized colloidal silica	0359AC
Choice 2	Bisco, Inc. Schaumburg IL, USA	Conventional	Bis-GMA	> 90% Strontium glass, Amorphous silica	1300005010
N-heptane	Daejung Chemical & metal CO., Ltd, Korea	pH= 6.8 Heptane 97%	NA	NA	h2827me1
Distilled Water	NA	pH= 7	NA	NA	NA
Apple juice	Alifard Co., (P.J.S)/ Kaveh Industrial city, Saveh, I.R.I.	pH= 3.5	NA	NA	4103023074355
Listerine mouth wash	Johnson & Johnson Brazil Health Products Industry and Commerce Ltd., Sao Jose dos Campos, SP, Brazil.	pH= 5 Menthol 0.042% thymol 0.064% methyl salicylate 0.06% Eucalypto10.092% Ethanol 21.6%	NA	NA	3400LZ

For the completion of an additional acid-base setting reaction, the specimens were stored at room tempera-ture for 24 h. Prior to removing the specimens from the mold, the excess material was removed through gentle wet manual grinding on both sides by using 1200-grit silicon carbide paper (Tufbak waterproof sand-ing sheets; Scour Pads Pty. Ltd., Vic, Australia). Then, the specimens were removed from the mold by gentle bending movement. Polymerized specimens were placed in a desiccator (Desiccator Glass; LabX Company, Ontario, Canada) with freshly dried silica gel (SIGMA-ALDRICH; Taufkirchen, Germany) main-tained at 37±1℃. After 24 hours, the specimens were removed and weighed to an accuracy of 0.0001 g by using an analytical scale (Ohaus Corporation; New Jersey, 07058, USA). This 24-hour desiccation cycle was repeated until a constant mass (m1) was obtained after 3 days (mass variation was less than ±0.01 mg). The diameter and thickness were measured using a digital caliper with accuracy up to 0.1 mm (Mini Electronic Caliper; Zhejiang, China). The mean diameter was calculated by measuring the diameter of each specimen at two points; these diameters were at right angles to each other. The mean thickness was cal-culated by measuring the thickness at five equally-spaced points on the circumference of the specimen. The volume (V) of each specimen was calculated in mm^3^ according to the equation

$$V=\pi \;(\frac{d}{2}{)}^{2}h$$

, where d is the diameter and h is the thickness of the specimen. For each material, 8 specimens were placed in a glass vial containing 40 ml of distilled water as the control group and 8 specimens were placed in a glass vial containing 40 ml of either Listerine mouthwash or food-simulating fluids (Table 1) as treat-ment groups. The vials were wrapped in aluminum foil to exclude light and were placed in an incubator at 37℃. The weight of the specimens was recorded every 24 h until a constant weight was achieved (m_2_) after two weeks. After each 24 h, the specimens were removed from the solutions, gently wiped with a soft paper towel to remove the excess solutions, weighed and immediately returned into the solution.

At the end of the immersion period, the specimens were placed in the desiccator following the desiccation procedure mentioned previously, until the specimens reached the constant mass (m_3_) after two weeks. Wa-ter sorption (Wsp

$$Wsp=\frac{m_{2}-m_{3}}{V}$$

) and Water solubility (Wsl,

$$Wsl=\frac{m_{1}-m_{3}}{V}$$

) for each specimen were calculated in µg/mm^3^ .In these equations, m_1_ is the specimen mass before im-mersion in solution, m_2_ is the specimen mass after immersion in solution, m_3_ is the specimen mass after the second desiccation procedure and V is the specimen volume.

**Statistical Analysis**

The data were statistically analyzed by SPSS software package (version 18; SPSS Inc., Chicago, IL, USA). Descriptive statistics and the means of the measurements with 95% confidence interval were used to illustrate the results. Two-way analysis of variance (ANOVA) was conducted to show a possible interaction between the materials and solutions. One-way ANOVA and Tukey’s test were used to show and compare the effect of solutions on each material. 

## Results

The means and standard deviations for sorption and solubility are shown in Table 2. For both sorption and solubility, the two-way ANOVA test showed a significant interaction between the solution and material (*p*< 0.05). Therefore, the effect of solution differed between the materials; each material performed differently between solutions. 

With the exception of seT and Clearfil SA Cement, the type of solution had a statistically significant effect on the sorption in all materials (*p*≤ 0.05). Sorption was higher in Listerine when affected Panavia F, and in apple juice when it was in contact with Choice 2. Although apple juice caused the highest sorption for seT and Clearfil SA in comparison with other media, the effect was not significantly different. Among the solutions, heptane and distilled water affected the materials significantly, *p*< 0.001, and *p*< 0.037 respectively, while the effect of apple juice and Listerine was not significant, *p*< 0.109 and *p*< 0.226, respectively. Among the materials, seT showed the highest water sorption in all solutions; while, Choice 2 showed the lowest. For solubility, the significant interaction between materials and solutions was even stronger (*p*< 0.002) than that of sorption (*p*< 0.05). The solubility was significantly higher in apple juice for Panavia F (25.0) and seT (33.5), in heptane for Clearfil SA (14.5), and significantly lower in Listerine for Choice 2 (-19.8). In distilled water, the differences in solubility means were significant for Panavia F and Choice 2 compared with seT and Clearfil SA. SeT and Clearfil SA showed the lowest solubility (0.00) followed by Panavia F (8.0) and Choice 2 (8.5). The difference between Listerine and distilled water was statistically significant for Choice 2 (-19.8), insignificant but higher for Panavia F (20.5), and equal for seT and Clearfil SA (0.0). 

**Table 2:** Mean sorption and solubility values (µg/mm[3]) ± (SD) obtained for resin luting cements in various solutions

**Property**	**Material**	**Distilled water**	**Heptane**	**Apple juice**	**Listerine mouth wash**
Sorption	Panavia F	61.3± (29)^ba1^	29.3± (11)^b1^	37.6± (23)^ba13^	65.16± (15)^a1^
	Choice 2	18.5± (7)^ab2^	0± (0)^d1^	25± (0)^a13^	14.6± (6)^b2^
	SeT	95± (17)^a3^	77.8± (48)^a2^	114± (36)^a23^	65± (21)^a1^
	Clearfil SA Cement	29.16± (13)^a2^	31.3± (13)^a1^	67.3± (55)^a23^	39.6± (12)^a2^
Solubility	Panavia F	8± (6)^a1^	12.3± (11)^ab1^	25± (8)^b1^	20.5± (15)^ab1^
	Choice 2	8.5± (4)^a1^	0± (0)^ab12^	-7.2± (6)^b2^	-19.8± (7)^c2^
	SeT	0± (0)^b2^	19± (10)^a13^	33.5± (18)^a13^	0± (0)^b3^
	Clearfil SA Cement	0± (0)^b2^	14.5± (5)^a13^	11.4± (3)^a1^	0± (0)^b3^

Letters show differences between the solutions for each material (row) and numbers show differences between the materials in the same solution (column) (*p*≤ 0.05).

## Discussion

Clinically, resin luting cements are indicated when greater retention is needed[11] and are used for cementation of ceramic restorations as they increase the durability of the cemented ceramics.[2] Since the cements are inevitably exposed to oral fluids, they should withstand deterioration after exposure to changes in the oral environment. Cement degradation has been attributed to marginal leakage, hypersensitivity, secondary caries, and loss of restoration retention.[12] Degradation of materials in the oral cavity is composed of two components; mechanical and chemical.[10] It is reasonable to assume that the chemical component initiates the resin cement degradation.[13] In this study, four different immersion solutions were selected to simulate the oral environment alterations to which the restorative substrate is exposed.

According to the results of the present study, there existed significant differences in the water sorption and solubility values among the tested materials and solutions. Therefore, the null hypothesis was rejected. The observed difference might be due to the differences in resin cement composition and the surrounding media. The detected high sorption for the seT resin cement was in accordance with the findings of previous investigations. Mese *et al.* showed that seT had the highest sorption in both water and ethanol in comparison with the other cements.[4] The increased sorption of seT is very likely to be due to its relatively less filler contents and greater resin matrix portion in comparison to the other tested cements (Table 2). Resin-based materials with lower filler content also displayed higher sorption.[14] Furthermore, cements with greater matrix portion are more prone to hydrolysis and subsequent degradation.[15] On the contrary, the sorption and solubility of Choice 2 were less than all other cements, which could be attributed to its higher filler loading (78.9 Wt%) than the other tested resin cements.[16] In addition, the self-adhesive cements are produced with hydrophilic acidic monomers such as carboxylic acid or phosphoric acid groups, which will make the cement more susceptible to sorption and subsequent solubility.[17] This might explain the relatively high sorption values for seT and Clearfil SA cements. Variation in the amount of acidic monomers influences the sorption and solubility and is likely to be the cause of detected difference between these two cements. However, due to the variability in cement reactions in different solutions, this observation should be confirmed by an additional research.

In terms of marginal adaptation, ceramic restorations exhibited more marginal opening than the restorations with metal fitting surfaces.[18] The implications of marginal opening are greater exposure for cementation material to oral environment which could directly increase the amount of deterioration. Therefore, it is reasonable to employ a resin luting cement with low sorption and solubility for cementing all ceramic restorations. A direct relation was reported between water sorption and solubility of dental resin; the solubility increased as water sorption increased.[19]

This study illustrated the significant effect of surrounding media on the cement solubility and sorption. Acidic environments such as citric acid and ascorbic acid of apple juice appeared to be harsh environments and clearly contributed to the greater solubility of resin cement. This confirms the findings of several studies on the effect of low pH.[3, 5] For example, Marghalani found that immersion of resin cements in lactic acid increased the sorption and solubility.[3] The negative effect of acid is attributed to the vulnerability of resin cement matrix to hydrolysis after being exposed to hydrogen ions.[15] The presence of hydrogen ion accelerates the catalysis for the ester groups of dimethacrylate monomers.[20] It causes degradation of the polymer crosslinking and softens the resin cement.[20] Eventually, monomer release is enhanced and with prolonged acidic exposure, the external filler particles are released from the cement mass. Although the solubility of resin cement appears to increase in acidic medium, Yoshida *et al.* and Hamouda reported that resin cements still exhibit significantly less solubility than the conventional acid-base cements.[5, 21] Therefore, the clinical significance of acidic deterioration is yet to be determined since constant exposure of exposed resin cement to acid in oral environment is very unlikely due to the continuous protective buffering capacity.

Alcohol-containing solution appears to have some effect on cement sorption and solubility. This supports Toledano *et al.*’s study that showed alcohol-containing solutions have increased the solubility of self-cured resin composite.[22] Likewise, Moraes Porto *et al.* showed that Listerine mouthwash could increase the solubility in two tested composite resins.[23] This was attributed to the efficiency of ethanol as the solvent of resin crosslinking networks.[24-26] Ethanol can easily penetrate the resin matrix and cause swelling and release of unreacted monomers.[27] As unreacted monomer is likely to remain within the cement mass, it is vulnerable to be dissolved by the solvent. Hand-mixing of resin material may incorporate air voids that can induce inhibition of resin polymerization; thus, increase the amount of monomer and subsequent solubility.[7, 28] Furthermore, the porosity enhances the transportation of fluid through the cement, the subsequent swelling and crosslinking dissolution.[20] Panavia, for being a type of hand-mixed cement, could exhibit greater solubility than the other cements in alcohol-containing solution. Since the study design differed from the clinical application of resin cements, the methodology is expected to be responsible for the obtained outcome. Disc-shaped specimens in constant medium are more susceptible to degradation due to constant effect of the solution on a large surface and when compared to minimally-exposed cement at the margin of restoration, the magnitude of sorption and solubility would likely be less.

As proposed by earlier researches, this study used heptane as fatty food simulator.[29-30] Generally, heptane solution was found to increase the sorption and solubility of most cements. This confirms the previous researchers’ findings.[29-30] The effect of heptane solution was attributed to the ease of penetration into resin matrix[29] and the subsequent plasticizing effect.[30] The susceptibility of resin matrix to softening can explain why Choice 2 was the least affected by heptane solution.

It must be emphasized that there was a prominent variability in the solubility values ranging from negative to positive values. The significant variation could be a result of the experimental set-up of this study. All materials were subjected to some, but variable, degrees of dissolution after immersion. The reason of different mass changes after the second desiccation could be related to the equilibrium of water uptake and actual mass loss which was different for each material. Some of the absorbed water was firmly bounded to the resin matrix and could not be completely removed.[31] Therefore, after the desiccation process which followed water storage, only that amount of water which was loosely connected to the matrix was removed. As each material behaves differently in each condition, the overall readings differ.

The outcome of this study should be carefully interpreted, since the clinical presentation is more complex. Indeed, the clinical excessive cement exposure is normally restricted to the restoration margin which is about 100 µm. Clinical recommendations can be provided following the clinical evaluation of resin cement materials. The studies on solubility and sorption mainly examine the integrity of cement in terms of relationship of the filler content and the resin matrix with the surrounding environment. However, clinically, the failure of the cemented restoration frequently occurs at the cement-tooth or cement-restoration interfaces, rather than within the cement layer.[32-33]

## Conclusion

Within the limitations of this study, a significant material-dependent interaction was detected between the solution and material (*p*< 0.05). 

Among the solutions, heptane and distilled water affected the materials significantly. Among the materials, seT showed the highest sorption in all solution; while, Choice 2 showed the lowest.

For solubility, the significant interaction between the materials and solutions was even stronger (*p*< 0.002) than that of sorption (*p*< 0.05). In distilled water, the differences in solubility means were significantly higher for Choice 2 and Panavia F compared with seT and Clearfil SA. 
